# Common Transcriptomic Effects of Abatacept and Other DMARDs on Rheumatoid Arthritis Synovial Tissue

**DOI:** 10.3389/fimmu.2021.724895

**Published:** 2021-08-30

**Authors:** Clement Triaille, Patrick Durez, Tatiana Sokolova, Gaëlle Tilman, Laurent Méric de Bellefon, Christine Galant, Pierre Coulie, Bernard R. Lauwerys, Nisha Limaye

**Affiliations:** ^1^Pôle de pathologies rhumatismales systémiques et inflammatoires, Institut de Recherche Expérimentale et Clinique, Université catholique de Louvain, Brussels, Belgium; ^2^Service d’Hématologie, Oncologie et Rhumatologie pédiatrique, Cliniques Universitaires Saint-Luc, Brussels, Belgium; ^3^Service de Rhumatologie, Cliniques Universitaires Saint-Luc, Brussels, Belgium; ^4^Service d’Anatomie Pathologique, Cliniques Universitaires Saint-Luc, Brussels, Belgium; ^5^de Duve Institute, Université catholique de Louvain, Brussels, Belgium; ^6^Genetics of Autoimmune Diseases and Cancer, de Duve Institute, Université catholique de Louvain, Brussels, Belgium

**Keywords:** synovial biopsy, transcriptomic profiling, abatacept, disease modifying anti-rheumatic drug, synovitis, treatment response, rheumatoid arthritis

## Abstract

**Objectives:**

Our goal was to assess for the histological and transcriptomic effects of abatacept on RA synovia, and to compare them with previously published data from four other DMARDs: tocilizumab, rituximab, methotrexate, and adalimumab.

**Methods:**

Synovial tissue was obtained using ultrasound-guided biopsy from affected joints of 14 patients, before and 16 weeks after treatment with subcutaneous abatacept 125 mg weekly. Paraffin-sections were stained and scored for CD3^+^, CD20^+^, and CD68^+^ cell infiltration. Transcriptional profiling was performed using GeneChip Human Genome U133 Plus 2.0 arrays (Affymetrix) and analyzed on Genespring GX (Agilent). Pathway analyses were performed on Genespring GX, Metascape, and EnrichR.

**Results:**

Gene expression analysis identified 304 transcripts modulated by abatacept in synovial tissue. Downregulated genes were significantly enriched for immune processes, strongly overlapping with our findings on other therapies. Data were pooled across these studies, revealing that genes downregulated by DMARDs are significantly enriched for both T-cell and myeloid leukocyte activation pathways. Interestingly, DMARDs seem to have coordinate effects on the two pathways, with a stronger impact in good responders to therapy as compared to moderate and non-responders.

**Conclusion:**

We provide evidence that the effects of five DMARDs on the RA synovium culminate in the same pathways. This confirms previous studies suggesting the existence of common mediators downstream of DMARDs, independent of their primary targets.

## Introduction

Despite the increasing arsenal of targeted therapies used to treat rheumatoid arthritis (RA), a significant proportion of patients fail to reach clinical remission (1, 2). Consequently, the use of alternating cycles of different therapies after insufficient response is not uncommon. Notwithstanding their disparate primary targets, cytokines/cytokine receptors (TNFα, IL6/IL6R), cell populations (CD20^+^ B cells), co-stimulatory molecules (CD80/86), or signaling proteins (Janus Kinases; JAKs), biological/targeted synthetic disease-modifying anti-rheumatic drugs (b/tsDMARDs) show strikingly similar levels of efficacy in large-scale studies patients refractory to methotrexate (3, 4). Furthermore, the probability of response to any particular DMARD decreases with the number of DMARDs previously used, regardless of class (drug-target) switch (3–5). These clinical observations led to the hypothesis that DMARDs have convergent effects downstream of their immediate targets (the *common pathway hypothesis*). This is supported by a series of studies from our group showing that different DMARDs induce similar transcriptomic changes in paired (pre- *versus* post-treatment) RA synovial biopsies (6–8).

As the major site of disease, the synovium is now widely recognized to provide an unparalleled view of both pathological and therapeutic processes operating in RA (9–12). Here, we use global transcriptomic profiling in association with clinical, ultrasonographic, and immunohistochemical evaluation of synovial tissue, before and after abatacept (CTLA4Ig) initiation. We find abatacept mainly modulates lymphocyte-related transcripts (T Cell-related genes and chemokines). By combining these data with those generated from four other DMARDs: methotrexate, tocilizumab, rituximab, and adalimumab (6–8), we provide compelling evidence, in a large series (50 pre-/post-treatment pairs) of synovial biopsies, for a shared set of highly inter-connected genes and pathways modulated downstream of RA therapies.

## Material and Methods

### Patients and Samples

Fourteen patients with active RA despite methotrexate treatment were included in the study (**Table 1**). All patients met the ACR/EULAR 2010 RA classification criteria. The study was approved by the ethics committee of the Université catholique de Louvain (2017/15NOV/515). All patients gave written inform consent to participate in the study.

**Table 1:** Baseline characteristics of patients (n = 14) included in the study.

Age in years (median ± SD)	57.2 ± 14.4
Female	9/14
Disease duration in years (median ± SD)	11.7 ± 8.1
RF and/or ACPA seropositivity	8/14
Erosive disease	12/14
Baseline disease activity (DAS28CRP) (median ± SD)	4.78 ± 1.11
*High*	5/14
*Moderate*	8/14
*Low*	1/14
EULAR response at W16	
*Good*	6/14
*Moderate*	4/14
*None*	4/14
Ongoing treatment	
cDMARD	14/14
Prednisone (<10 mg/d)	3/14
Previous bDMARD use	0/14
Biopsy localization	
*Wrist*	8/14
*Metatarsophalangeal joint*	3/14
*Metacarpophalangeal joint*	1/14
*Knee*	2/14

ACPA, Anti Citrullinated Protein Antibody; bDMARD, Biological Disease-Modifying Anti-Rheumatic Drug; cDMARD, Classical Disease-Modifying Anti-Rheumatic Drug; SD, Standard Deviation; RF, Rheumatoid Factor.

For each patient, synovial biopsies were obtained from the same affected joint before (W0) and 16 weeks after (W16) starting treatment with abatacept 125mg subcutaneously per week. Clinical response according to EULAR criteria was assessed at the time of 2^nd^ biopsy. Six to 10 synovial biopsy fragments were obtained using ultrasound-guided biopsy. Half were stored at -80°C after overnight incubation in RNA-Later solution (Invitrogen). The rest were fixed overnight in 10% formalin buffer at pH 7.0 and embedded in paraffin for histology and immunohistochemistry.

Data from four other cohorts of RA patients with active disease, included in previous studies on pre/post treatment biopsies (6–8), were also analyzed: 2 × 8 patients treated with adalimumab (baseline cDMARD 8/8, 2 good responders (GR), 4 moderate responders (MR), and 2 non responders (MR); EULAR response criteria), 2 × 12 patients treated with rituximab (baseline cDMARD 12/12, 3 GR, 6 MR and 3 NR), 2 × 8 biopsies from patients treated with methotrexate (baseline cDMARD 0/8, 2 GR, 2 MR and 4 NR), and 2 × 12 patients treated with tocilizumab (baseline cDMARD 0/12, 7 GR, 4 MR and 1 NR).

### Ultrasonographic Assessment

Ultrasonographic (US) scoring of the biopsied joint was performed at W0 and W16. Briefly, an experienced rheumatologist (LMDB) assigned each biopsied joint a score for hyperplasia on gray-scale (US GS). US GS score ranges from 0 (no detectable hyperplasia) to 3 (severe hyperplasia).

### Transcriptional Profiling

Total RNA was extracted from synovial biopsies using Tripure Isolation Reagent (Roche) after mechanical disruption with an Ultra-turrax (Sigma Aldrich). RNA quality was assessed using an Agilent 2100 Bioanalyzer and RNA nanochips. Complementary RNA (cRNA) was synthesized from 100 ng total RNA, and biotin-labelled according to a standard Affymetrix procedure (GeneChip 3′ IVT Plus). GeneChip Human Genome U133 Plus 2.0 arrays were hybridized overnight at 45°C with 10 μg fragmented biotinylated cRNA. The slides were then washed and stained using a EukGE‐WS2v5 fluidics protocol on a GeneChip Fluidics Station 450, before being scanned on a GeneChip Scanner 3000 (Affymetrix). The Affymetrix.CEL files were deposited in the Gene Expression Omnibus of the National Center for Biotechnology Information, and are accessible through Gene Expression Omnibus accession number GSE172188.

### Immunohistochemical Analyses

Immunolabeling experiments were performed using a standard protocol, as previously described (8). The following antibodies were used: anti‐CD3 (Neomarkers), anti‐CD20 (Biocare Medical), and anti‐CD68 (DakoCytomation). Evaluation of immune cell infiltration was performed by an expert pathologist (CG) blinded to clinical data, using a semiquantitative score on a 0–3 scale, where 0 indicates absence of the feature and 3 represents the highest level.

### Statistical Analyses

Analyses of microarray data were performed using GeneSpring GX software (Agilent). Fluorescence intensity data were normalized using robust multiarray analysis (RMA). For the independent analysis of each treatment, pre/post treatment differentially expressed genes (DEG) were calculated using paired Mann-Whitney test (uncorrected p-value threshold <0.05). For the pooled analysis of all treatments, intensity data per transcript per sample were first normalized within each experiment, and normalized intensity values from all samples were then collapsed into a single dataset. Pre/post treatment DEG were calculated using paired Mann-Whitney test (Benjamini-Hochberg corrected *p*-value threshold <0.05). Log_2_ fold change (FC) in expression were calculated on Genespring GX. Samples from each of the five cohorts were classified as low (“L”) or high (“H”) for *T Cell* and *Myeloid Leukocyte Activation* signatures at baseline using unsupervised clustering (Median linking rule, Canberra metric) based on the level of expression of the genes in these pathways. Principal component analysis (PCA) was performed on Genespring GX. Geneset enrichment analyses were performed on Genespring GX, Metascape (https://metascape.org/gp/index.html#/main/step1) and EnrichR (https://maayanlab.cloud/Enrichr/) (13, 14). Circos plots were generated using Metascape. Protein-Protein Interaction (PPI) network analysis was performed on STRING webtool (https://string-db.org/) (15). All other statistical analyses were performed on Graphpad Prism v9.

## Results

### I. Effects of Abatacept on the RA Synovium: Clinical, Immunohistochemical, and Transcriptomic Indices

**Figure 1:**
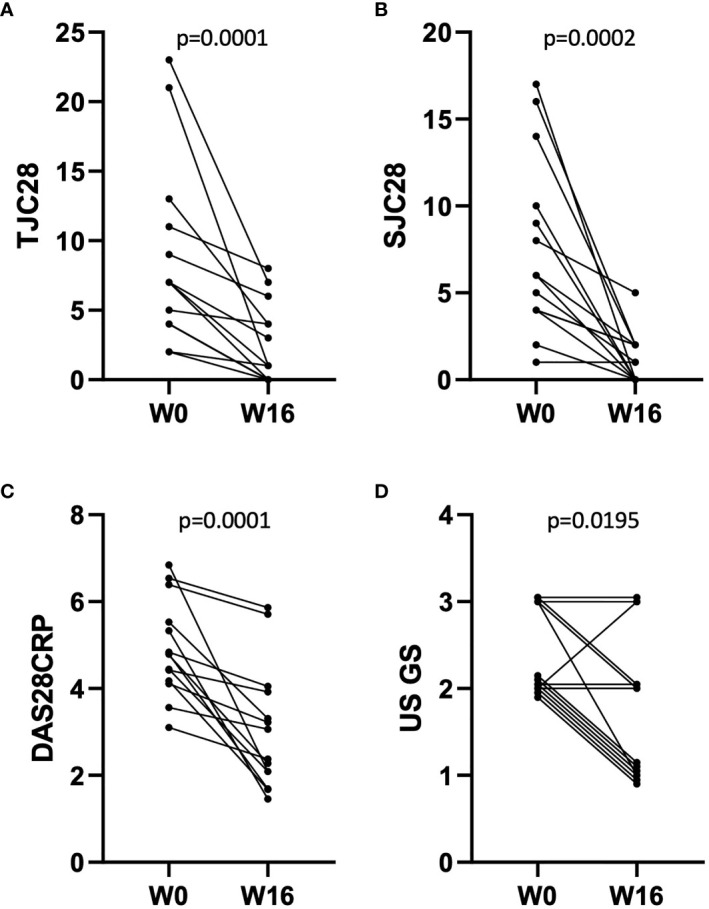
Evolution of disease activity in n = 14 patients between W0 and W16 of treatment with abatacept. **(A–D)** Effect of abatacept on TJC28 **(A)**, SJC28 **(B)**, DAS28CRP **(C),** and US gray-scale score **(D)**. p-values: Wilcoxon matched-pairs ranked test. Overlapping points are offset for clarity of representation in **(D)**.

Synovial biopsies were collected from fourteen methotrexate-resistant RA patients (median disease duration 11.7 years, ACPA/RF positivity: 57%, erosive disease: 86%), before and 16 weeks after treatment with abatacept 125 mg per week subcutaneously. A summary of patient and sample characteristics is provided in **Table 1**. RNA from both pre- and post-treatment biopsies could be obtained for 10 patients, and paired histological sections for 11. Clinical assessment showed a significant effect of abatacept on disease activity (**Figures 1A–D**): mean TJC28, SJC28, DAS28CRP decreased by 70.6%, 83.9%, and 37.8%, respectively, between W0 and W16. Overall, 7/14 patients reached remission (DAS28CRP < 2.6) at W16.

Immunohistochemical evaluation of immune cell infiltration (semi-quantitative CD3^+^, CD20^+^ and CD68^+^ scores) did not show any significant differences between pre- and post-abatacept treatment biopsies (**Supplementary Figures S1A–C**), possibly due to the number of samples (6 out of 11) that were lymphocyte poor (CD3^+^/CD20^+^ scores ≤0.5; **Supplementary Figures S1A, B**) at baseline, i.e., pre-treatment. Response to abatacept (EULAR response, % remission) did not differ between the baseline lymphocyte-rich *vs*. lymphocyte-poor groups. As previously described (16), scores for the different cell-types showed a high degree of correlation across all (n = 22) samples, as did their changes between W0 and W16 (**Supplementary Figures S1D–G**).

**Figure 2:**
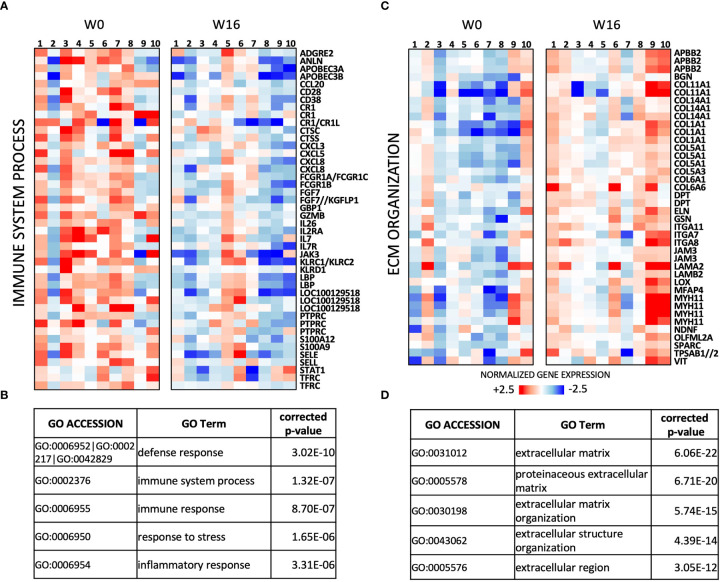
Transcriptomic effects of abatacept in n = 10 pairs of pre/post-treatment RA synovial biopsies. **(A)** Heat-map of relative expression of *Immune System process* genes downregulated by abatacept (FC ≥ 1.5, p < 0.05, paired Mann-Whitney). **(B)** Top 5 Gene Ontology (GO) terms enriched in 129 transcripts downregulated between W0 and W16 (FC ≥ 1.5; p < 0.05, paired Mann-Whitney). **(C)** Heat-map of relative expression of *extracellular matrix* (*ECM)* genes upregulated by abatacept (FC ≥ 1.5, p < 0.05, paired Mann-Whitney). **(D)** Top 5 GO terms enriched in 175 transcripts upregulated between W 0 and W 16 (FC ≥ 1.5; p < 0.05, paired Mann-Whitney).

A total of 304 transcripts showed differential expression (fold change (FC) ≥ 1.5, uncorrected p < 0.05; **Supplementary Table S1**) between the paired post- and pre-treatment samples in this series. Genes downregulated by abatacept (n = 129 transcripts) are mainly involved in immune response, and include key regulators of T-cell activation (e.g. *IL2RA*, *CD28*, *IL7*, and *IL7R*) as well as chemokines (**Figures 2A, B**). The 175 transcripts upregulated in post-treatment RA synovia are enriched for extracellular matrix (ECM) organization, possibly reflecting tissue repair and remodeling (**Figures 2C, D**).

### II. Common Transcriptomic Effects of DMARDs in RA Synovial Tissue

**Figure 3:**
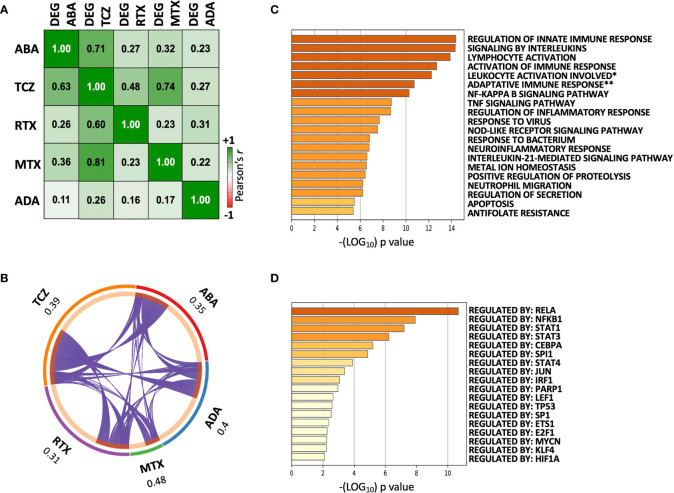
Overlapping transcriptomic effects of five DMARDs on paired pre/post-treatment RA synovial biopsies. **(A)** Correlation matrix showing Pearson’s *r* between mean Log_2_FCs induced by pairs of DMARDs. Cells show values for the differentially expressed genes of each DMARD (Columns: “DEG”), *vs*. the modulation of these same transcripts by each of the other DMARDs (Rows). “DEG ABA” = 5824 transcripts significantly modulated by ABA between W0 and W16, “DEG TCZ” = 6726 transcripts, “DEG RTX” = 4577 transcripts, “DEG MTX” = 1228 transcripts, “DEG ADA” = 3383 transcripts. p < 10e^-8^ for all correlations. **(B)** Circos plot showing overlap between genes downregulated by each DMARD, and all other DMARDs. Each purple line joins a pair of shared genes. For each DMARD, the fraction of unique genes downregulated by at least one other DMARD is indicated. **(C)** Top 20 pathways identified by enrichment analysis of 227 genes downregulated by ≥3/5 DMARDs. Full pathway IDs: *Leukocyte activation involved in immune response, **Adaptive immune response based on somatic recombination of immune receptors built from immunoglobulin superfamily domains. **(D)** Transcription factor enrichment analysis of 227 genes downregulated by ≥3/5 DMARDs.

We next wished to evaluate whether abatacept shows overlapping effects with other DMARDs, particularly those that also target lymphocytes. We harnessed previously published transcriptomic data from our group, generated using the same (paired synovial biopsy) experimental design, to compare: abatacept (ABA, n = 10 × 2 biopsies), methotrexate (MTX, n = 8 × 2 biopsies) (8), tocilizumab (TCZ, n = 12 × 2 biopsies) (8), rituximab (RTX, n = 12 × 2 biopsies) (7), and adalimumab (ADA, n = 8 × 2 biopsies) (6). The transcriptional effects (fold-changes post- *vs*. pre-treatment) of several DMARDs were significantly correlated (**Figure 3A**). TCZ, which, like ABA, includes T cells amongst its primary targets, showed the greatest concordance with ABA-modulated DEGs (Pearson r = 0.71). As previously, TNF-blocking therapy (ADA) showed the least similarity to all other therapies. We compared the lists of genes significantly downregulated by each DMARD and found considerable overlap: 31–48% of genes downregulated by a particular DMARD were also downregulated by at least one other (**Figure 3B**). Pathway analysis of genes downregulated by three or more (of the five) DMARDs showed enrichment for innate and adaptive immune processes known to play critical roles in RA pathophysiology (**Figure 3C**); transcriptional factor (TF) enrichment analysis showed concordant results, with over-representation of genes regulated by the NF-κB complex and STATs (**Figure 3D**). A similar degree of overlap was also observed for genes upregulated by DMARDs (**Supplementary Figure S2A**); pathway analysis showed weaker enrichment of genes, in heterogeneous processes including tissue morphogenesis and ossification (**Supplementary Figure S2B**).

**Figure 4:**
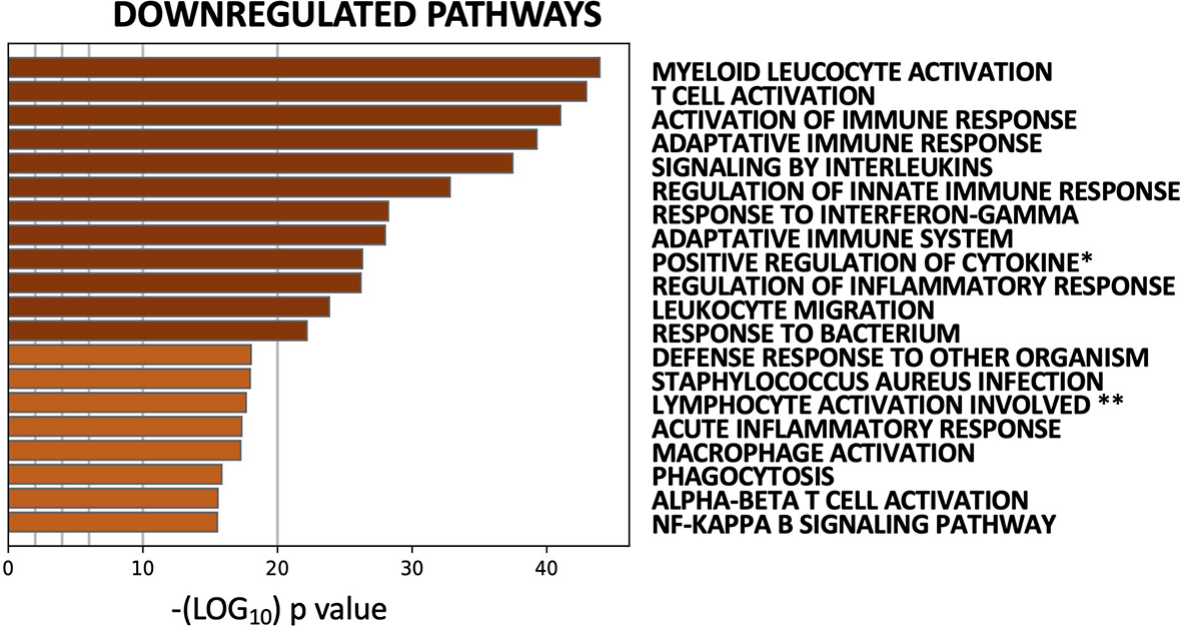
Common transcriptomic effects of five DMARDS identified by pooled analysis of 50 paired pre-/post-treatment RA synovial biopsies. Top 20 pathways identified by enrichment analysis of 573 genes downregulated by DMARDs (paired Mann-Whitney with Benjamini-Hochberg corrected p-value <0.05). Full pathway IDs: *Positive regulation of cytokine production. **Lymphocyte activation involved in immune response.

The striking coherence in transcriptional modulation by multiple DMARDs led us to pool data from all five cohorts into a single analysis (50 pre/post treatment pairs), to assess for significant common effects. Downregulated (573) and upregulated (574) genes (Benjamini-Hochberg corrected p-value <0.05) were identified in post- *vs*. pre-treatment biopsies collapsed across treatments (**Supplementary Table S2**). Pathway analysis of upregulated genes showed modest enrichment for processes related to skeletal system development and morphogenesis (**Supplementary Figure S3A**). Downregulated genes, on the other hand, were strongly enriched for both *Myeloid Leukocyte Activation* and *T Cell Activation* **(** **Figure 4**, **Supplementary Figure S3B**). PPI network analysis on downregulated genes showed high inter-connectivity, with hub proteins including LCK, STAT1, STAT3, JAK2, and JAK3 (**Supplementary Figure S4A**); accordingly, they were enriched for targets of JAK inhibitors (**Supplementary Figure S4B**). Intriguingly, downregulation of this gene network was significantly associated with clinical (EULAR) response (**Supplementary Figure S4C**), supporting its relevance to and reflection of RA pathogenesis.

**Figure 5:**
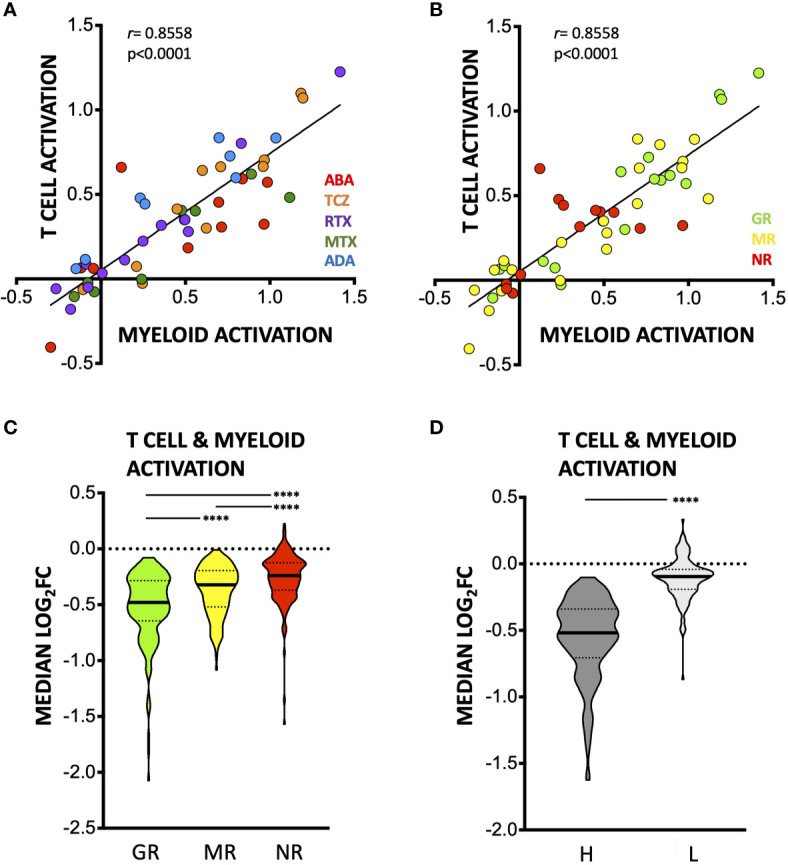
Effects of DMARDs on *T-cell* and *Myeloid Leukocyte Activation* transcriptional signatures. **(A, B)** Median – (log_2_FC) in n = 80 T Cell activation genes (as defined by Metascape) and n = 38 myeloid activation genes (as defined by Metascape), colored by therapy **(A)** or by clinical response at time of 2^nd^ biopsy **(B)**. **(C)** Truncated violin plot (with median and interquartile range) of median log_2_FC in expression of (n = 118) T-cell and myeloid cell activation genes induced by DMARDs in groups with different EULAR response at 2^nd^ biopsy. ****p < 0.0001, Kruskal-Wallis with Dunn’s multiple comparison tests. GR, good responders; MR, moderate responders; NR, non-responders. **(D)** Truncated violin plot (with median and interquartile range) showing median log_2_FC in expression of T-cell and myeloid cell activation genes induced by DMARDs in samples classified as high (dark grey, n = 33) or low (light grey, n = 17) for T-cell and myeloid cell signatures (n = 118 genes) at baseline by unsupervised clustering (see Methods). ****p < 0.0001, Mann-Whitney test.

Transcriptional signatures of myeloid *versus* lymphoid activation have been proposed to represent distinct processes in the synovium (11, 17), with the former postulated to be preferentially modulated by TNF-blockade (ADA) and the latter by TCZ (17). We did not detect therapy-specific effects on the genes in either process, modulation of which was well-correlated with each other (**Figure 5A**, **Supplementary Figure S5** and **Supplementary Table S3**). Degree of downregulation was instead associated with clinical response (**Figures 5B, C**, **Supplementary Figure S6A**) and pre-treatment expression levels of these genes (**Figure 5D**; **Supplementary Figures S6**, **S7**). Indeed, the myeloid activation and T-cell activation modules were significantly more downregulated in good EULAR responders (GR, n = 17) as compared to moderate (MR, n = 20) and non-responders (NR, n = 13), across therapies (**Figures 5B, C**). This strongly suggests coordinate effects of DMARDs on both arms, baseline levels of which are broadly associated with response to these therapies.

## Discussion

Increasing evidence shows that the in-depth analysis of RA synovial tissue delivers relevant information on pathogenic mechanisms and therapeutic modes of action (9, 10). We assessed for the effects of abatacept on RA synovitis by comparing pairs of pre/post treatment synovial biopsies from 14 patients. Abatacept was found to downregulate the expression of immune genes including key T-cell regulators, while upregulating genes involved ECM organization.

We found no significant reduction in T cell, B cell, and macrophage infiltration (by immunohistochemistry) after abatacept. This may be partly due to the number of low-inflammatory baseline samples in our series. A second issue may be reduced representativeness of immunohistochemistry (a more limited, two-dimensional field) as compared to bulk transcriptional profiling that assesses the entire tissue. Finally, it is possible that abatacept blocks T cell-antigen presenting cell crosstalk without necessarily reducing absolute cell numbers. In line with this, a previous study on abatacept also reported no effects on T-cell and macrophage synovial infiltration, and only a modest effect on B cell numbers (18).

Comparing abatacept with four other DMARDs, we found significant overlap in their transcriptional effects, with post-treatment biopsies showing significant downregulation of the *Myeloid Leukocyte* and *T Cell Activation* pathways. This despite inter-cohort differences, including ongoing MTX treatment in some cohorts (ABA, ADA, and RTX), which may have impacted baseline transcriptomes. Interestingly, clinical response was correlated with the degree of downregulation of genes in these pathways; greater downregulation was also observed in samples with higher baseline expression of these genes. This suggests that a high baseline immune activation signature in the synovium may be a broad predictor of response to DMARDs. This is in line with previous observations showing that low-inflammatory synovitis is associated with poor response to bDMARDs targeting TNFα (19). Nevertheless, it is unclear whether low-inflammatory synovitis represents a distinct disease (sub) entity, or simply an extreme of a phenotypic continuum (11, 12, 20, 21).

Predominant lymphoid *versus* myeloid immune features have been proposed to distinguish between independent, even disparate entities in RA (11, 17). The innate and adaptive arms of immunity are however highly inter-dependent, not least in RA pathogenesis: CD4^+^ T cells (either as T peripheral helpers in ectopic lymphoid structures or as central memory T cells) activate macrophages, fibroblast-like synoviocytes (FLS), and B cells (4, 22). Reciprocally, macrophages, B cells, and FLS that express HLA Class II act as antigen-presenting cells and produce immunomodulatory cytokines, playing key roles in synovial cross-talk (4, 23). The high degree of interconnectivity makes it implausible for one or more of these participants to act independently, without ripple effects upon other, intersecting processes. Accordingly, we show that DMARDs seem to have coordinate (rather than mutually exclusive) effects on the T and myeloid cell signatures in RA synovium: no matter their immediate/primary targets and proximal effects, they culminate in a largely overlapping pattern of transcriptional changes. TF analysis and PPI-network analyses suggest JAK/STATs may lie at the crossroads of their actions. Immunohistochemistry-based studies have previously shown shared, response-dependent effects of different DMARDs in RA synovium, mostly on myeloid cells. Thus, treatment-induced decrease in CD68^+^ cell infiltration in the sub-lining is correlated with clinical response to several drugs with different modes of action (24, 25). Using tissue transcriptomics, we now show that modulation of T cells is also associated with response to different therapies.

The limitations of this study are intrinsically related to the use of bulk transcriptomic profiling of heterogeneous tissue which primarily reflect changes in gene expression of predominant cell populations; changes in (numerically) minor cell-types can be lost. A more granular view (single-cell or spatial transcriptomic profiling of paired biopsies) could potentially reveal distinct effects of DMARDs on less abundant cell-types, in addition to common denominators modulated in highly-represented cells. By capturing and distinguishing between their effects on different cell populations, such approaches may also provide more mechanistic insights into pathological and therapeutic processes in RA. Our studies nevertheless suggest that downregulation of T-cell and myeloid cell activation are well-correlated with one another, and associated with treatment response across DMARDs that target different cell populations and signaling pathways.

## Data Availability Statement

The datasets presented in this study can be found in online repositories. The names of the repository/repositories and accession number(s) can be found below: https://www.ncbi.nlm.nih.gov/geo/, GSE172188.

## Ethics Statement

The studies involving human participants were reviewed and approved by ethics committee of the Université catholique de Louvain. The patients/participants provided their written informed consent to participate in this study.

## Author Contributions

BL and PD designed the study. PD, TS, LM, and CG collected the biological samples and clinical data. CT, GT, BL, and NL took part in the experimental procedures. CT, PC, BL, and NL analyzed and interpreted the data. All authors wrote and revised the manuscript. All authors contributed to the article and approved the submitted version.

## Funding

This work was funded in part by unrestricted grants from Cap48 (RTBF) and Bristol-Myers Squibb, the Fonds de la Recherche Scientifique (FNRS) under Grant no. CDR J.0138.20, and the Fund for Scientific Research in Rheumatology (FWRO/FRSR), managed by the King Baudouin Foundation (Grant no. 2019-J5820590-214283). CT is funded by the FNRS and Fondation Saint-Luc (Cliniques Universitaires Saint-Luc). NL is a chercheur qualifiée of the FNRS.

## Conflict of Interest

BL is currently employed at UCB Biopharma.

The remaining authors declare that the research was conducted in the absence of any commercial or financial relationships that could be construed as a potential conflict of interest.

## Publisher’s Note

All claims expressed in this article are solely those of the authors and do not necessarily represent those of their affiliated organizations, or those of the publisher, the editors and the reviewers. Any product that may be evaluated in this article, or claim that may be made by its manufacturer, is not guaranteed or endorsed by the publisher.

## Supplementary Material

Supplementary Figure 1 **(A–C)** Evolution of CD3 score **(A)**, CD20 score **(B)**, and CD68 score **(C)** between W0 and W16. Grey dashed lines indicate cutoff score of 0.5. W0 *vs*. W16 not statistically significant (Wilcoxon matched-pairs ranked test). **(D)** Correlation of W16-W0 difference in CD3 scores and W16-W0 difference in CD20 scores. **(E–G)** Correlation of individual CD3, CD20, and CD68 scores in n = 22 samples. Overlapping points are offset for clarity of representation.

Supplementary Figure 2 **(A)** Circos plot showing overlap between genes upregulated by each DMARD, and all other DMARDs. Each purple line connects a pair of shared genes. For each DMARD, the fraction of unique genes upregulated by at least one other DMARD is indicated. **(B)** Top 20 pathways identified by enrichment analysis of genes upregulated by ≥3/5 DMARDs.

Supplementary Figure 3 **(A)** Pathway enrichment analysis (top 100) of 574 genes upregulated by DMARDs in pooled analysis. **(B)** Pathway enrichment analysis (top 100) of 573 genes downregulated by DMARDs in pooled analysis.

Supplementary Figure 4 **(A)** STRING protein–protein interaction (PPI) network of 573 genes downregulated by DMARDs in pooled analysis. Only highest confidence interactions are shown. Unconnected nodes are removed. Network statistics indicated in figure. Red stars: top 10 hub proteins on EnrichR (LCK, STAT1, STAT3, JAK2, and JAK3, shown; PTPN6, LYN, CBL, SYK, and FYN not shown as absent in DMARD-downregulated gene list). **(B)** Enrichment of kinase-inhibitor targets on KinomeScan (*via* EnrichR) amongst 573 DMARD-downregulated genes. **(C)** Truncated violin plot (with median and interquartile range) showing median log_2_FC (post- *vs*. pre-treatment) in expression of 702 transcripts, in groups with different EULAR response at second biopsy. ******p < 0.0001, Kruskal-Wallis with Dunn’s multiple comparisons test. GR: good responders, MR: moderate responders, NR: non-responders.

Supplementary Figure 5 Correlation matrix showing Pearson’s coefficient between DMARD-induced log_2_FC in genes involved in T-cell activation (n = 80 genes) and myeloid leukocyte activation (n = 38 genes).

Supplementary Figure 6 **(A)** Principal components analysis (PCA) on Log_2_FC in T-cell and myeloid leukocyte activation genes. PC1 accounts for 26.05% of variance and PC2 for 9.01%. Samples are colored according to EULAR response (GR: good responders, MR: moderate responders, NR: non-responders). Black outline: samples with a low baseline expression of T-cell and myeloid leukocyte activation genes (Methods & **Supplementary Figure S7**). **(B)** Number of patients in each category of EULAR response to therapy and baseline level of expression of T-cell activation and myeloid leukocyte activation genes (H = High, L = Low; Methods & **Supplementary Figure S7**).

Supplementary Figure 7 **(A–E)** Unsupervised hierarchical clustering (Canberra metric, median linking rule) of baseline (pre-treatment) samples from five independent series: ABA samples **(A)**, TCZ samples **(B)**, RTX samples **(C)**, MTX samples **(D)**, and ADA samples **(E)**, based on expression of genes in T-cell and myeloid cell activation pathways (n = 199 genes; **Supplementary Table S3**). Black boxes: samples with low baseline expression of these genes (“L”; n = 17 total).
