# Supplementary material for: Common Transcriptomic Effects of Abatacept and Other DMARDs on Rheumatoid Arthritis Synovial Tissue
Source: Front Immunol. 2021 Aug 30;12:724895. doi: 10.3389/fimmu.2021.724895 (PMC8435834; doi:10.3389/fimmu.2021.724895)

Supplementary Figure 1

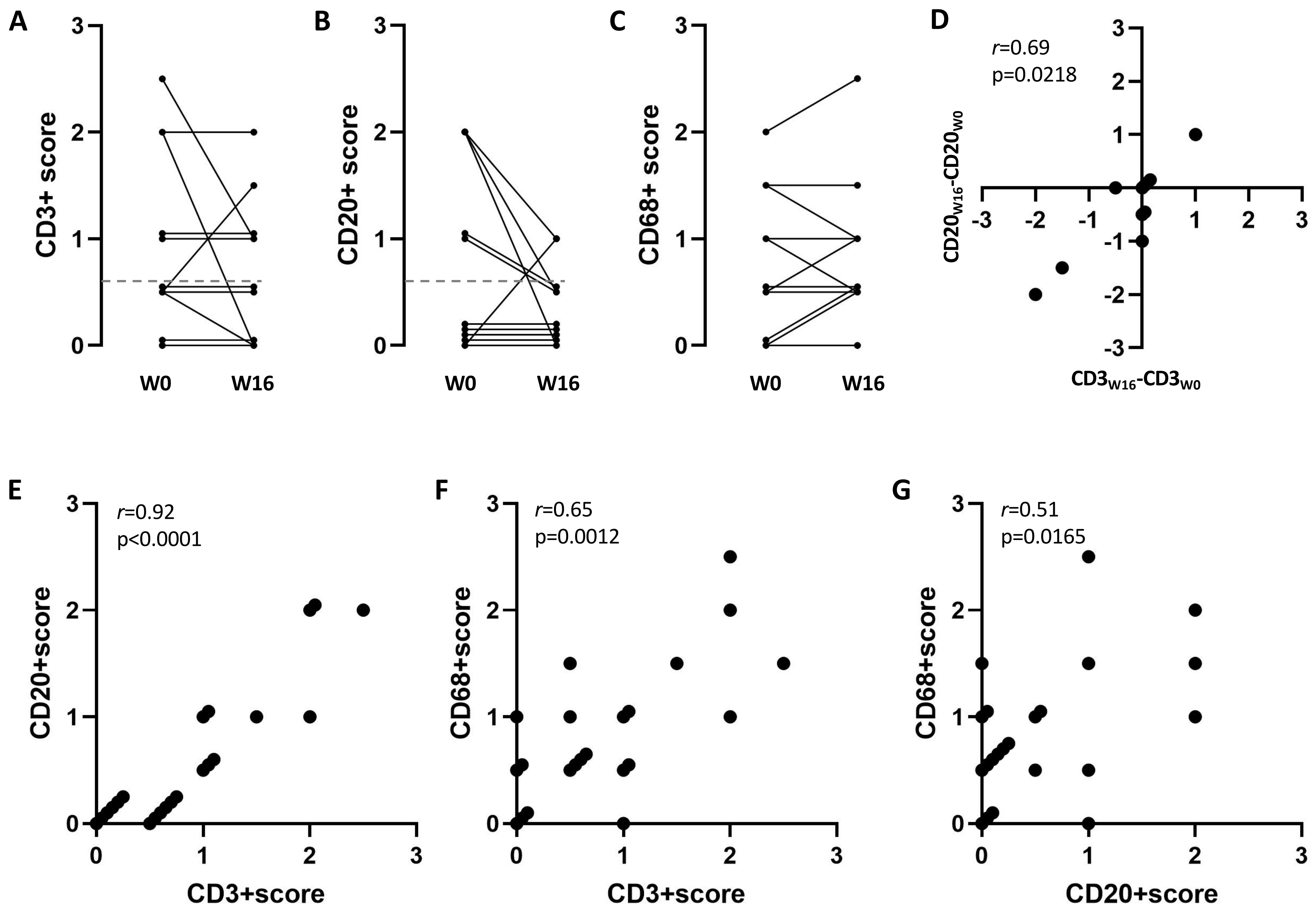

Supplementary Figure 2

A

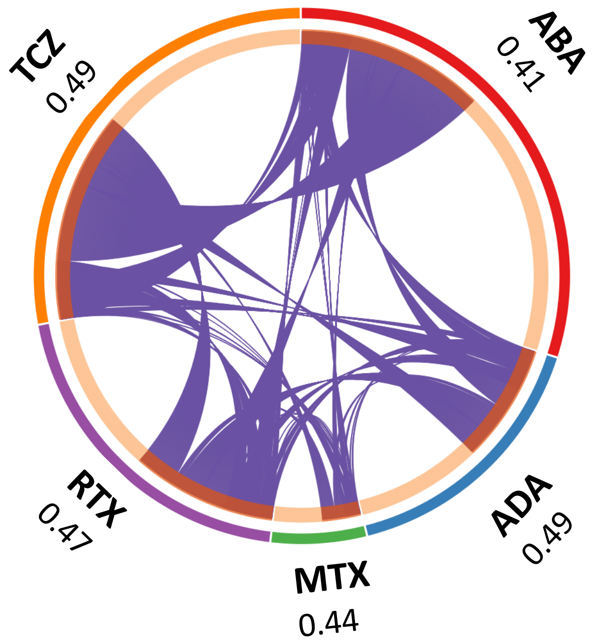

B

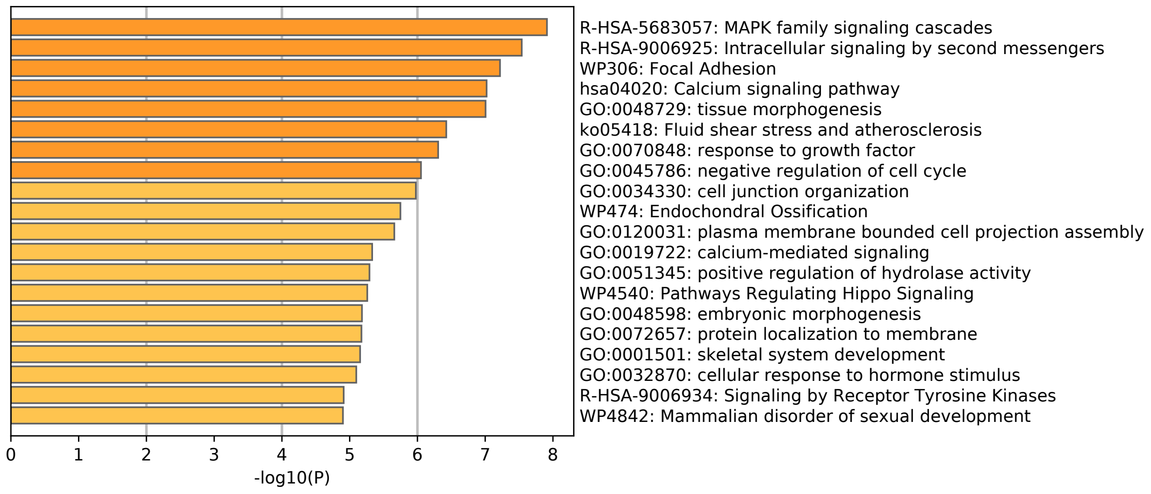

Supplementary Figure 3

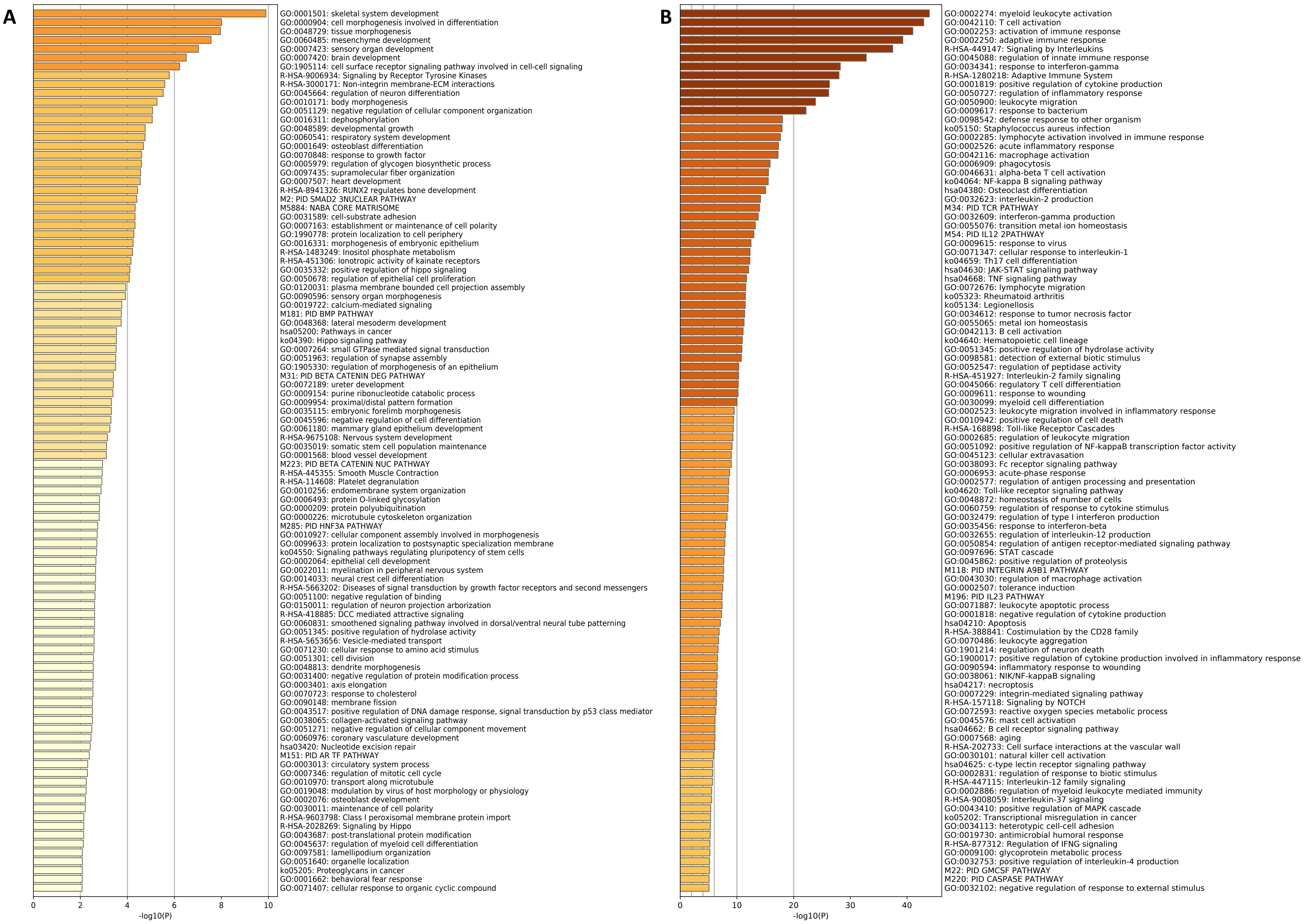

Supplementary Figure 4

A

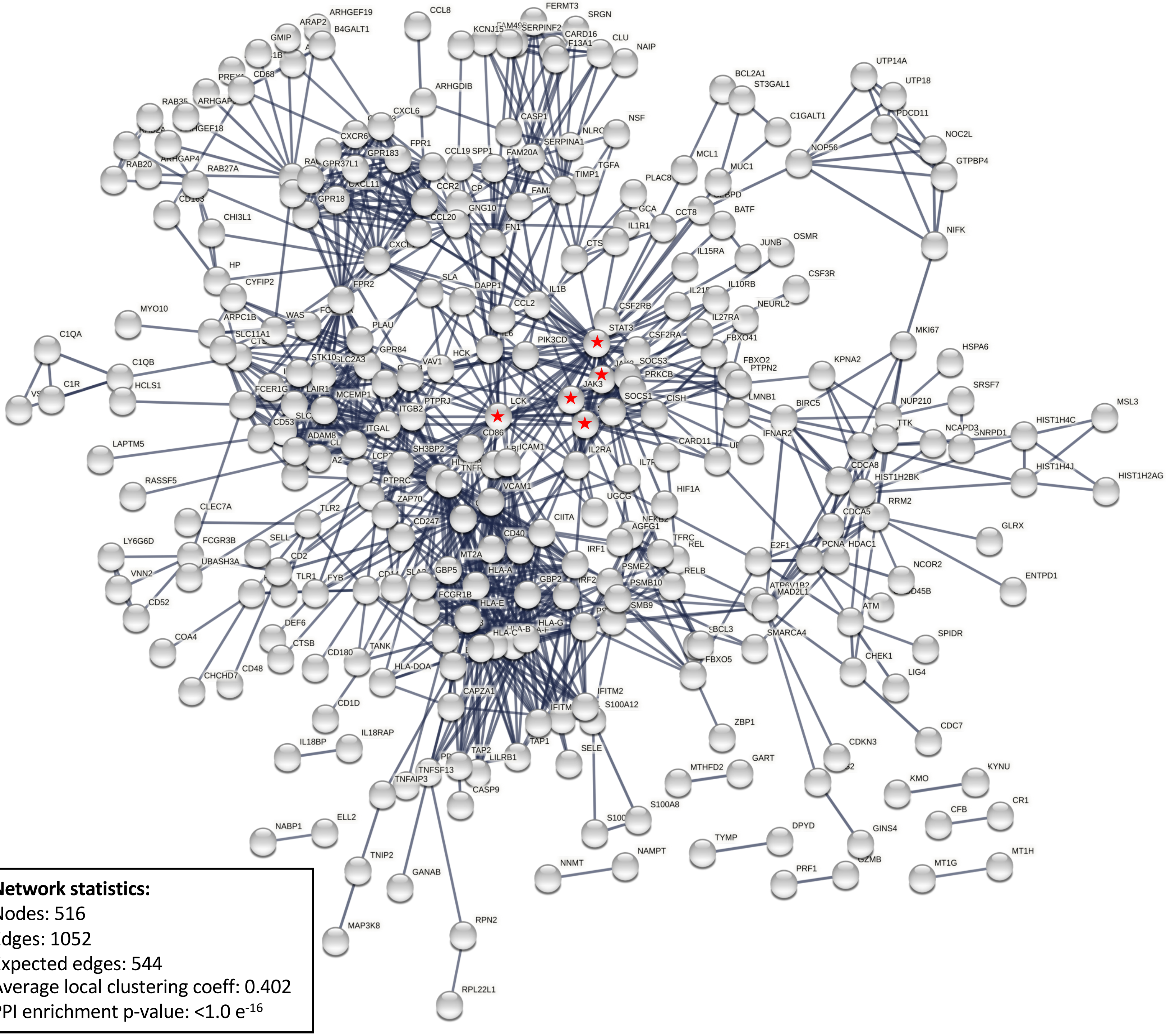

Network statistics:
Nodes: 516
Edges: 1052
Expected edges: 544
Average local clustering coeff: 0.402
PPI enrichment p-value: <1.0 e^{-16}

Supplementary Figure 4

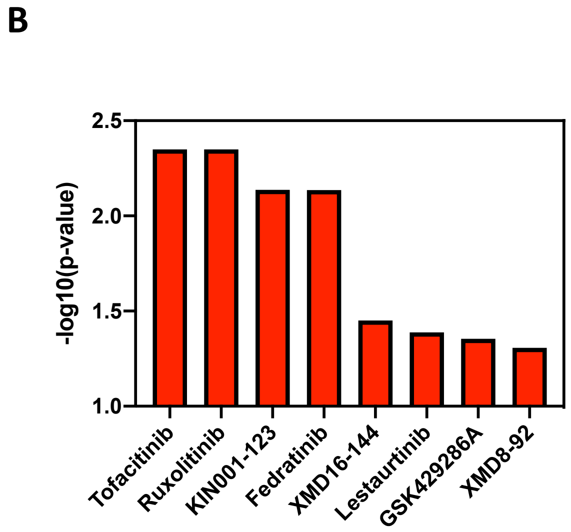

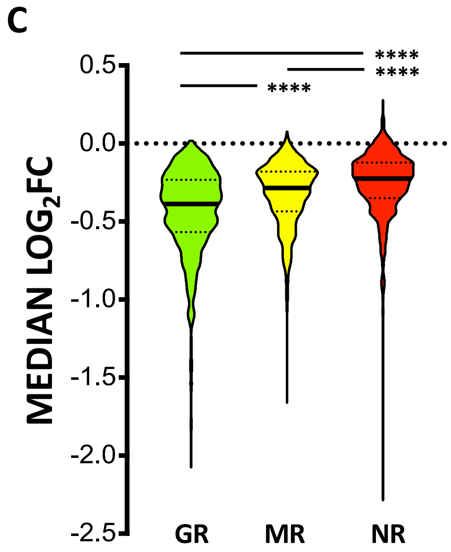

Supplementary Figure 5

A

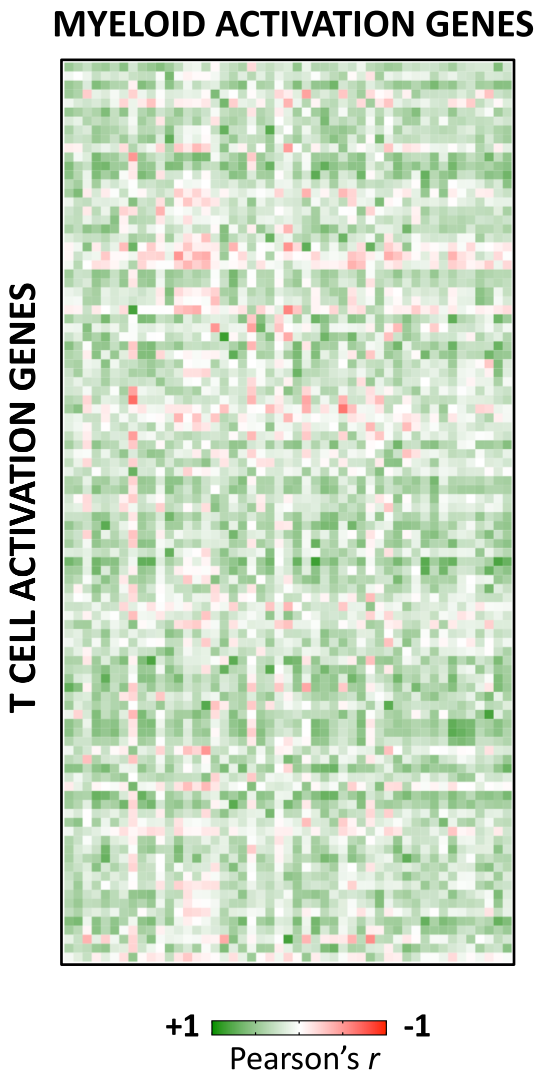

Supplementary Figure 6

A

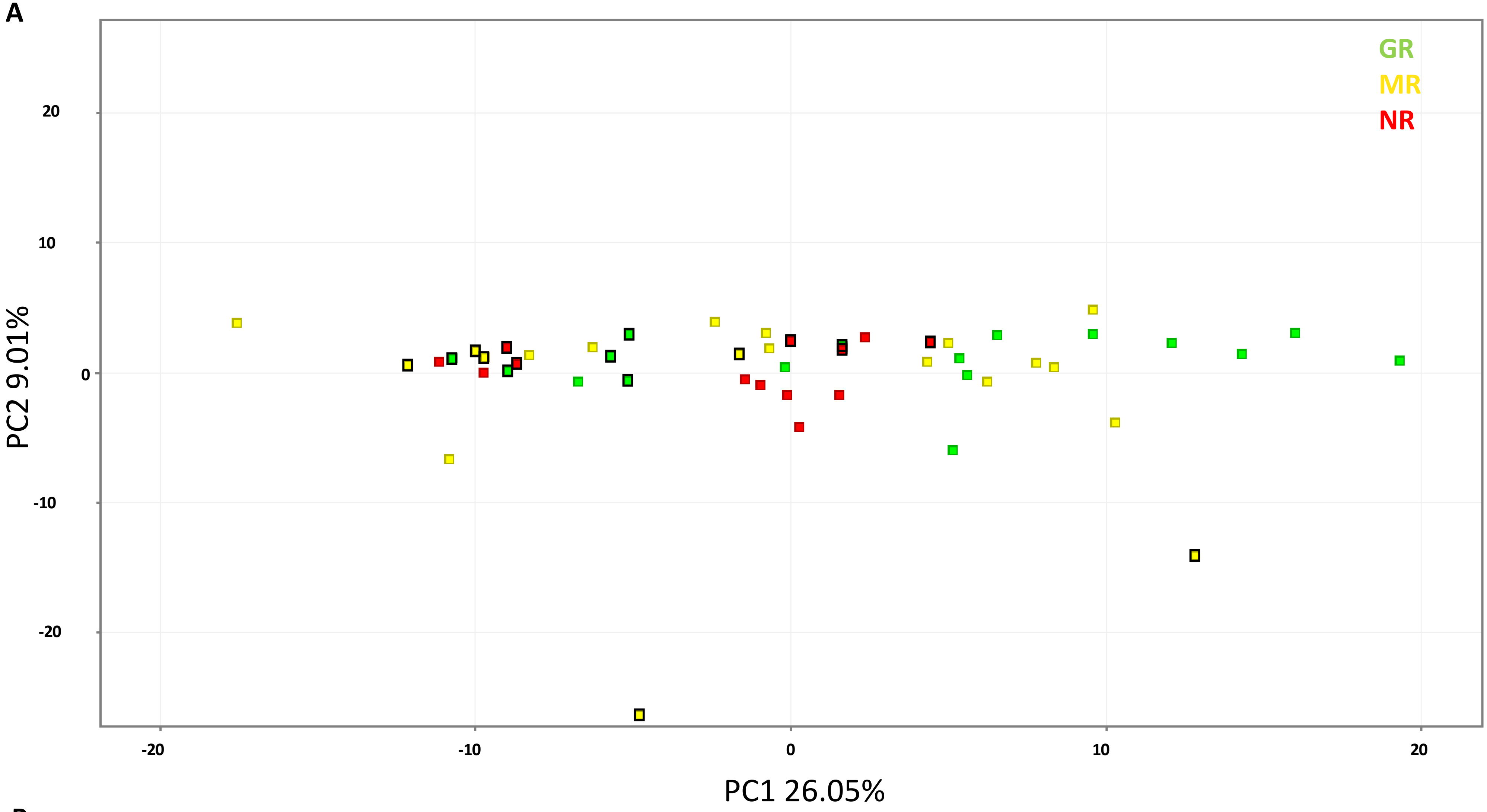

B

n of patients	H	L
**GR**	11	6
**MR**	14	6
**NR**	8	5

Supplementary Figure 7

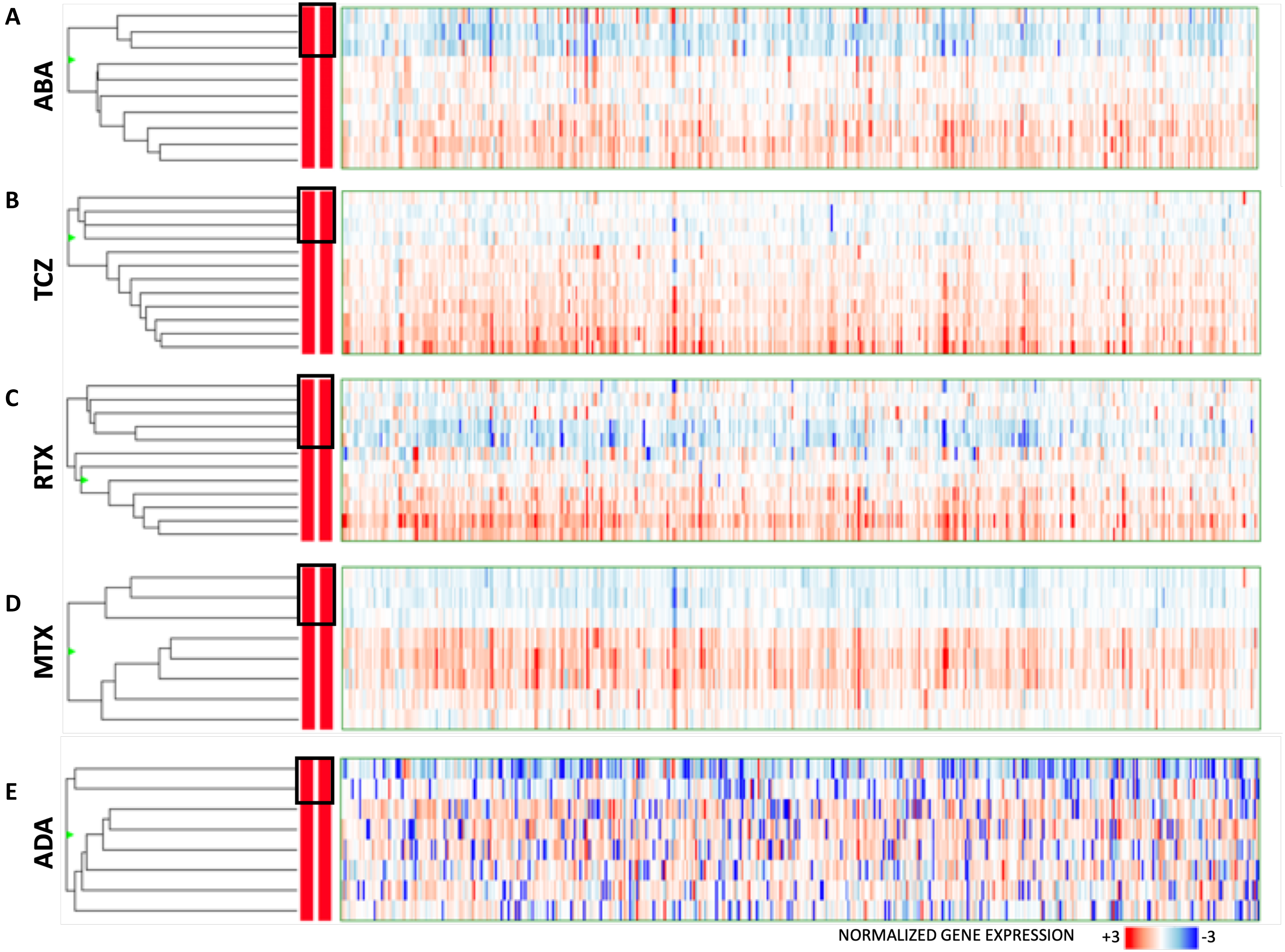

Supplement: Supplementary Figure 1 — (A–C) Evolution of CD3 score (A), CD20 score (B), and CD68 score (C) between W0 and W16. Grey dashed lines indicate cutoff score of 0.5. W0 vs. W16 not statistically significant (Wilcoxon matched-pairs ranked test). (D) Correlation of W16-W0 difference in CD3 scores and W16-W0 difference in CD20 scores. (E–G) Correlation of individual CD3, CD20, and CD68 scores in n = 22 samples. Overlapping points are offset for clarity of representation. [file DataSheet_1.pdf]
